# A Three-Year Analysis of Mortality in Clostridioides difficile Patients in a Tertiary Center

**DOI:** 10.7759/cureus.74291

**Published:** 2024-11-23

**Authors:** George S Gherlan, Simin Aysel Florescu, Mihaly Enyedi, Ion Cristian Efrem, Adina Mitrea, Diana Clenciu, Stefan D Lazar

**Affiliations:** 1 Infectious Diseases, Clinic Hospital of Tropical and Infection Diseases "Dr. Victor Babes", Bucharest, ROU; 2 Infectious Diseases, University of Medicine and Pharmacy "Carol Davila", Bucharest, ROU; 3 Anatomy, University of Medicine and Pharmacy "Carol Davila", Bucharest, ROU; 4 Internal Medicine and Gastroenterology, University of Medicine and Pharmacy of Craiova, Craiova, ROU; 5 Diabetes and Endocrinology, University of Medicine and Pharmacy of Craiova, Craiova, ROU

**Keywords:** clostridioides difficile, death, diarrhea, mortality, unfavourable prognostic

## Abstract

Background/objectives: *Clostridioides difficile*, an anaerobic bacillus ubiquitous in nature, is the leading cause of hospital-acquired diarrhoea and one of the main causes of mortality by nosocomial infections. We aimed to identify the main predictors of the risk of dying and the characteristics of a three-year cohort of patients hospitalised in our clinic that eventually had an unfavourable outcome.

Methods: We collected retrospectively available data for all patients hospitalised between January 1, 2021, and December 31, 2023. The characteristics of the patients who died after the CDI (*Clostridioides difficile *infection) were analysed and compared with those of the patients who survived.

Results: In the three-year interval mentioned above, 1086 patients had the main or secondary diagnosis of CDI. Of these, 97 patients (8.93%) died. The overall mortality for the same period was 2.62%. Eight patients (8.24%) who died had the primary diagnosis of CDI, while in the entire group, the percentage of patients with a primary diagnosis was 54.7%. Statistically significant differences between the groups of deceased and survivor patients were found for the following parameters: age (p<0.001, 95% CI (confidence interval): 12.5-20.5), previous CDI episodes (p=0.033, 95% CI: 0.014-0.329), and for the following parameters measured at admission: systolic blood pressure, quick sepsis-related organ failure assessment (qSOFA), leucocyte count, haemoglobin, creatinine, albumin, potassium, INR (international normalised ratio), CRP (C-reactive protein), fibrinogen, and procalcitonin. The number of hospitalisation days for the patients who died was significantly higher (p<0.001, 95% CI: 4.3-12.6.).

Conclusions: We identified the characteristics that significantly differentiated the patients who died from those who survived. Mortality is significantly higher in the group of patients with CDI than that in the other hospitalised patients.

## Introduction

*Clostridioides difficile* (CD), formerly known as *Clostridium difficile*, is an anaerobic Gram-positive bacillus able to sporulate, ubiquitous in nature, and recognised as the leading cause of hospital-acquired diarrhoea [1,2]. It was first identified in 1935 in the stool of 10 breast-fed newborns in their first days of life by Hall and O'Toole [3]. They named the newly discovered bacteria *Bacillus difficile* because it was difficult to isolate and culture, and they also showed that it produces a toxin that can be lethal to mice [4]. At this time, pseudomembranous colitis (PMC) had already been discovered since 1893, but no connection was made with *Bacillus difficile* until 1978, when George, Bartlett, and Larson described it in three different journals.

*Clostridioides difficile* infections (CDIs) are traditionally associated with healthcare contact but are increasingly diagnosed in patients without recent contact, thus showing community transmission. The emergence of an epidemic was first signalised in Canada in 2003, when, besides an alarming increase in the number of cases, an increase in severity was also described [5]. Similar patterns were identified soon in the USA and Europe [6-8]. The North American outbreak was probably caused by ribotype 27, the so-called North American pulse-field type 1 (NAP1) strain identified in 2005 and was shown to cause more severe forms of the disease and higher mortality than other strains [9].

CDI may manifest in various forms, from asymptomatic carriage to deadly forms. CDI can also be seen in non-severe, severe, and fulminant forms (FCDI) [10]. Asymptomatic carriage occurs in approximately 20% of hospitalised adult patients and may be as high as 50% in long-term care facilities [11]. According to the American College of Gastroenterology Guidelines, severe forms are defined by the presence of either one of leukocytosis (over 15000 cells/mmc) or increased creatinine (over 1.5 mg/dl) [12]. The European Society of Clinical Microbiology and Infectious Diseases adds to these criteria a fever above 38.5°C and sustaining arguments such as large intestine distension, colonic wall thickening, or pericolonic fat stranding at imaging [13]. Non-severe forms are those who do not have any of the above criteria, and fulminant forms are severe forms that associate hypotension, shock, ileus, megacolon, elevated serum lactate, bowel perforation, or any fulminant course of the disease [12,13]. Some unusual CDI presentations have been described, including protein-losing enteropathy with consecutive hypoalbuminemia in the absence of fulminant colitis [14], appendicitis due to CDI [15], and extracolonic localisation of CDI (small bowel enteritis, cellulitis due to CDI, reactive arthritis) [16].

Many studies have shown that hospitalised CDI patients tend to have more comorbidities, higher Charlson comorbidity indexes, and a higher risk of dying than patients hospitalised with any other comorbidities [17-21]. Mortality rates range from 6% to 11% in patients hospitalised in general departments [18,19] and can be as high as 37% in ICUs (intensive care units) [22]. CDI has also been proven to be an independent factor that increases the length of stay in the hospital and discharge to a care facility [23]. Classical risk factors for developing a severe form of CDI are older age, increased leucocyte count (over 15000 cells/mmc), increased creatinine (over 1.5 mg/dl), decreased albumin (below 3 g/dl), increased markers of inflammation (C-reactive protein (CRP)), and the use of antibiotics and proton pump inhibitors (PPI) [24,25]. The use of chemotherapy can also be incriminated as a risk factor for a severe or even deadly form [26,27].

Recurrent CDI (rCDI) also predicts a worse outcome [18,19]. Charlson's score for comorbidities is another tool that can indicate a severe or even deadly outcome [18,26,28]. The ATLAS score stratifies the chances to achieve cure by medical treatment in 11 classes (scores from 0 to 10); for score 0, the chances are 100%, while for score 10, 49.2% [29]. It uses five simple parameters measured at the time of diagnosis: age, serum creatinine, serum albumin, leucocyte count, and concomitant antibiotic use during CDI treatment, each given a score from 0 to 2, depending on their values.

Based on the above information, we decided to perform an analysis of the patients with CDI who were hospitalised in our institution. The primary objectives of the study were to assess the characteristics of the patients who died in our hospital after a CDI episode and compare them to those of the patients who survived, as well as to identify risk factors for death in patients suffering from CDI. The secondary objective of the study was to create a score to stratify the risk.

## Materials and methods

Study design

We conducted a retrospective study including all the patients hospitalised with the principal or secondary diagnosis of CDI in Dr. Victor Babes Clinical Hospital for Infectious and Tropical Diseases, which is a tertiary infectious diseases unit located in Bucharest, Romania, with a capacity of 490 beds.

Data collection

The collection process included the analysis of the data recorder for all the patients hospitalised in our institution with a CDI diagnosis. In contrast, the patients who did not present with this disorder were excluded from this analysis. In the case of patients who presented with recurrent infections, only the data from the last hospitalisation were included in the study. The collected data were as follows: age, sex, weight, height, vital signs at admission (weight (kg), height (cm), heart rate (/minute), blood pressure (BP, mmHg), arterial oxygen saturation (%)), previous episode history, previous antibiotic use history, previous contact with healthcare facilities, data regarding diagnostic tests (toxin A and/or B positivity), duration from debut to hospitalisation, maximum number of bowel movements/24 hours, comorbidities, concomitant medication, patient status regarding being institutionalised in a long-term care facility, laboratory values at admission (complete blood count (CBC), chemistry (alanin-amino transferase, creatinine, ionogram (Na, K), glycaemia, albumin, protein electrophoresis), coagulation (INR (international normalised ratio), fibrinogen), inflammation markers (CRP and procalcitonin)), blood cultures (if available), other cultures (urine or wounds) (if available), days to stool normalisation (according to Bristol scale, types 1-4), and the outcome of the patient (survived/died).

Information about baseline chronic comorbidities in our CDI patients was also collected. In our analysis, comorbidities were grouped into the following categories: cardiovascular diseases (coronary heart diseases, blood hypertension, cerebrovascular diseases, peripheral vascular diseases, heart failure, valvular heart disease, thrombotic disease), liver diseases (chronic hepatitis of various etiologies (viral, toxic, autoimmune, etc.), established liver cirrhosis, biliary tract diseases), pulmonary diseases (asthma, chronic obstructive pulmonary diseases, chronic bronchitis, bronchiectasis), chronic kidney disease (renal insufficiencies of various degrees and etiologies, including end-stage kidney disease with haemodialysis necessity), diabetes mellitus type1 or 2, obesity (defined as BMI higher than 30 kg/m^2^), active cancer (active cancer of any type, solid or haematologic, localised or with metastasis), and psychiatric diseases (all forms of dementia of any cause, various other psychiatric disorders (schizophrenia, mood disorders, comportment disorders, etc.)). We calculated the ATLAS and Charlson scores for each patient.

Statistical analysis

Statistical analysis was made using IBM SPSS Statistics for Windows, Version 20 (Released 2011; IBM Corp., Armonk, New York). Continuous variables are presented as mean, minimum, maximum, and standard deviation. An independent-sample t-test was performed to compare the means of each analysed parameter, using the outcome as the grouping variable (death/recovered). Levene's test for equality of variances was also performed for each pair of means. We created ROC (receiver operating characteristic) curves for the statistically significant parameters and noted those with an AUROC (area under the receiver operating characteristic) higher than 0.6. ROC curves were calculated for each parameter using outcome death/recovered as the state variable. To identify the statistically independent predictors of death in our CDI group, we used multivariate linear regression. Parameter selection was made using Pillai's trace multivariate test; p-values <0.05 were considered significant. Based on the four parameters we identified in our group as independent predictors of death, we made a score selecting cutoffs that showed the best balance between sensitivity and specificity.

## Results

In the period mentioned above, 1086 patients were hospitalised with CDI, of whom 97 died. This means a mortality rate of 8.93%, while the general mortality in our hospital for the same period was only 2.62%. Overall, 595 patients had CDI as the principal diagnostic (54.7%), but in the group of deceased patients, only eight (8.24%) had CDI as the principal diagnostic. Table 1 shows an overview of the characteristics of the deceased patients at admission.

**Table 1:** Characteristics of deceased patients. ATLAS Age, Leu, Alb, Crea, and ATB are the scores calculated for age, leucocytosis, albumin, creatinine, and antibiotic use. Systolic BP: systolic blood pressure; BMI: body mass index; qSOFA: quick sepsis-related organ failure assessment; ALT: alanine aminotransferase; EGFR: estimated glomerular filtration rate; INR: international normalised ratio; CRP: C-reactive protein

Parameter	Minimum	Maximum	Mean	Std. Deviation
Age (years)	51	97	78.60	9.61
BMI (kg/m^2^)	12.34	39.95	25.12	5.18
Previous episodes	0	3	1.34	0.5
Days before hospitalisation	0	30	5.99	5.604
Systolic BP (mmHg)	60	187	113	25
SaO_2_	82	99	95	3.3
qSOFA	0	2	0.63	0.672
Leucocytes (/mm^3^)	1400	74000	14661	10146
Haemoglobin (g/dl)	6.2	18.5	10.9	2.2
Platelets (/mm^3^)	20000	1201000	278515	159119
ALT (IU/ml)	4	231	30	35
Creatinine (mg/dl)	0.3	8.1	1.6	1.3
EGFR (ml/min/1.73m^2^)	6.00	226.00	62.74	48.28
Albumin (g/dl)	0.7	2.7	1.4	0.57
Potassium (mmol/l)	1.6	6.1	3.30	0.79
INR	1.0	3.1	1.4	0.4
CRP (mg/dl)	.2	44.1	12.2	8.0
Fibrinogen (mg/dl)	129	793	441	156
Procalcitonin (ng/ml)	0.04	76.36	5.50	12.30
Hospitalisation days	0	94	18.2	14.7
ATLAS age	0	2	1.6	0.5
ATLAS Leu	0	2	0.4	0.7
ATLAS Alb	0	2	1.3	0.8
ATLAS Crea	0	2	0.7	0.8
ATLAS ATB	0	2	1.4	0.8
ATLAS	1	9	5.6	1.6

The characteristics of patients who did not die are shown in Table 2.

**Table 2:** Characteristics of patients who did not die. ATLAS Age, Leu, Alb, Crea, and ATB are the scores calculated for age, leucocytosis, albumin, creatinine, and antibiotic use. Systolic BP: systolic blood pressure; BMI: body mass index; qSOFA: quick sepsis-related organ failure assessment; ALT: alanine aminotransferase; EGFR: estimated glomerular filtration rate; INR: international normalised ratio; CRP: C-reactive protein

Parameter	Minimum	Maximum	Mean	Std. Deviation
Age (years)	19	91	62.06	17.23
BMI (kg/m^2^)	11.89	41.77	25.12	5.18
Previous episodes	0	3	1.17	0.4
Days before hospitalisation	1	22	6.75	5.04
Systolic BP (mmHg)	60	180	122	20
SaO_2_	86	100	96	2.1
qSOFA	0	1	0.14	0.345
Leucocytes (/mm^3^)	1700	42300	11589	7221
Haemoglobin (g/dl)	8.5	16.2	12.1	1.7
Platelets (/mm^3^)	20000	734000	286468	130307
ALT (IU/ml)	7	92	27	15
Creatinin (mg/dl)	0.5	4.5	1.1	0.67
EGFR (ml/min/1.73m^2^)	12.37	174.22	76.09	37.11
Albumin (g/dl)	1.5	4.8	3.1	0.88
Potassium (mmol/l)	2.2	4.8	3.56	0.91
INR	0.98	2.32	1.21	0.22
CRP (mg/dl)	0.1	34.2	7.7	7.7
Fibrinogen (mg/dl)	165	788	498	137
Procalcitonin (ng/ml)	0.05	61.33	2.29	10.04
Hospitalisation days	2	34	9.73	6.77
ATLAS Age	0	2	1.0	0.8
ATLAS Leu	0	2	0.3	0.6
ATLAS Alb	0	2	1.5	0.9
ATLAS Crea	0	2	0.4	0.6
ATLAS ATB	0	2	1.3	0.9
ATLAS	1	9	4.4	1.6

The mean age in the deceased patients' group was 78.6 years (51-97), while in the group of survivors, the mean age was 62.06 years (19-91). The youngest deceased patient was 51 years old. The oldest survivor was 91 years old. The difference between the two groups was 16.54 years, which is statistically significant (p<0.001, 95% CI: 12.5-20.5). Regarding gender, there were 56 (57.7%) female patients in the deceased patients' group and 41 (42.3%) males, with percentages almost similar in the survivors group, respectively, 599 (60.6%) female patients versus 390 (39.4%) males.

When we analysed the history of CDI, we remarked that patients in both groups had between zero and three previous episodes; the median in the deceased patients' group was 1.34, while in the survivors' group, it was 1.17. The mean difference of 0.172 was statistically significant (p=0.033, 95% CI: 0.014-0.329). However, the analysis of the number of days from symptom onset and hospital admission between the two groups did not reach statistical significance (p=0.349).

Regarding clinical parameters, the mean BP was statistically significant between groups, with lower BP at admission indicating a worse outcome.

The qSOFA (quick sepsis-related organ failure assessment) score is used to identify patients with possible sepsis and uses three simple parameters: BP, mental status, and respiratory rate. In the group of patients who died, the maximum qSOFA score was 2, while in the other group, it was 1, the difference reaching statistical significance (p<0.001, 95% CI: 0.34-0.65).

We also analysed blood count parameters and observed statistically significant values between the groups for leucocytes (p=0.017, 95% CI: 551-5593) and haemoglobin (p<0.001, 95% CI: 0.67-1.8).

Both liver and kidney functions were evaluated in the study group, but only in the case of creatinine (p=0.001, 95% CI: 0.21-0.82) and estimated glomerular filtration rate (eGFR) (p=0.04, 95% CI: 0.53-26.1) we observe statistically significant different values. Among the parameters that presented statistically significant values between the groups, we noted serum albumin, serum potassium, and INR.

Inflammatory markers also presented statistically significant differences between the studied groups, with mean CRP statistically significantly higher in the patients that died (p<0.001, 95% CI: 2.26-6.71). The differences between the two groups are summarised in Table 3.

**Table 3:** Differences between the two groups and their statistical significance. The p-value is obtained using an independent-sample t-test; the t-value is the value of t from an independent-sample t-test. The F-value is obtained from Levene's test for equality of variances. Systolic BP: systolic blood pressure; qSOFA: quick sepsis-related organ failure assessment; ALT: alanine aminotransferase; EGFR: estimated glomerular filtration rate; INR: international normalised ratio; CRP: C-reactive protein

Parameter	Mean for Patients Who Died	Mean for Patients Who Survived	Difference	Statistical Significance (p)	t-value	F-value
Age (years)	78.60	62.06	16.54	<0.001	8.219	24.839
Previous episodes	1.34	1.17	0.17	0.033	2.154	13.19
Days before hospitalisation	5.99	6.75	-0.76	0.349	-0.940	0.333
Systolic BP (mmHg)	113	122	-9	0.018	-2.394	6.085
SaO_2_	95	96	-1	0.256	-3.636	12.475
qSOFA	0.63	0.14	0.49	<0.001	6.217	79.545
Leucocytes (/mm^3^)	14661	11589	3072	0.017	2.404	3.711
Haemoglobin (g/dl)	10.9	12.1	-1.2	<0.001	-4.260	2.800
Platelets (/mm^3^)	278515	286468	-7953	0.310	-1.018	0.209
ALT (IU/ml)	30	27	3	0.296	1.018	9.101
Creatinin (mg/dl)	1.6	1.1	0.5	0.001	3.381	18.823
EGFR (ml/min/1.73m^2^)	62.74	76.09	-13.35	0.04	-2.056	3.072
Albumin (g/dl)	1.4	3.1	-1.7	<0.001	-6.096	1.357
Potassium (mmol/l)	3.30	3.56	-0.26	0.03	-2.192	8.630
INR	1.4	1.21	0.19	<0.001	4.228	13.830
CRP (mg/dl)	12.2	7.7	4.5	<0.001	3.946	0.007
Fibrinogen (mg/dl)	441	498	-57	0.017	-2.408	1.998
Procalcitonin (ng/ml)	5.50	2.29	3.21	0.179	1.353	3.473
Hospitalisation days	18.2	9.73	8.47	<0.001	4.074	15.824
ATLAS	5.6	4.4	1.2	<0.001	5.022	0.248

We introduced in the multivariate analysis all the above parameters, with the outcome of death/recovered as the fixed parameter. The Pillai's trace test result was 0.261, statistically significant (p<0.001), F(6,115) = 6.785, showing a relationship between some of the tested parameters and the outcome. We also performed a Bonferroni correction. Multivariate regression found the following statistically significant parameters: age (p<0.001), INR (p<0.001), CRP (p=0.002), and creatinine (p=0.023). The results are shown in Table 4.

**Table 4:** Multivariate analysis results: tests of between-subjects effects. Df: degrees of freedom; F: F ratio; INR: international normalised ratio; CRP: C-reactive protein

Source	Dependent Variable	df	Mean Square	F	Significance
Death	Age	1	6898.68	42.568	<0.001
	Creatinine	1	5.386	3.819	0.023
	INR	1	2.643	17.880	<0.001
	CRP	1	623.504	9.566	0.002

The estimated marginal means for the significant parameters are presented in Table 5.

**Table 5:** Estimated marginal means for age, INR, creatinine, and CRP from the multivariate analysis. The fixed parameter is outcome death: yes/no. INR: international normalised ratio; CRP: C-reactive protein

Dependent Variable: Death		Mean	Std. Error	95% Confidence Interval	
				Lower Bound	Upper Bound
Age	Yes	78.598	1.293	76.043	81.153
	No	64.041	1.819	60.446	67.635
INR	Yes	1.496	0.039	1.419	1.573
	No	1.211	0.055	1.102	1.319
Creatinine	Yes	1.660	0.121	1.421	1.898
	No	1.253	0.170	0.918	1.588
CRP	Yes	12.282	0.820	10.662	13.903
	No	7.906	1.153	5.626	10.186

**Figure 1:**
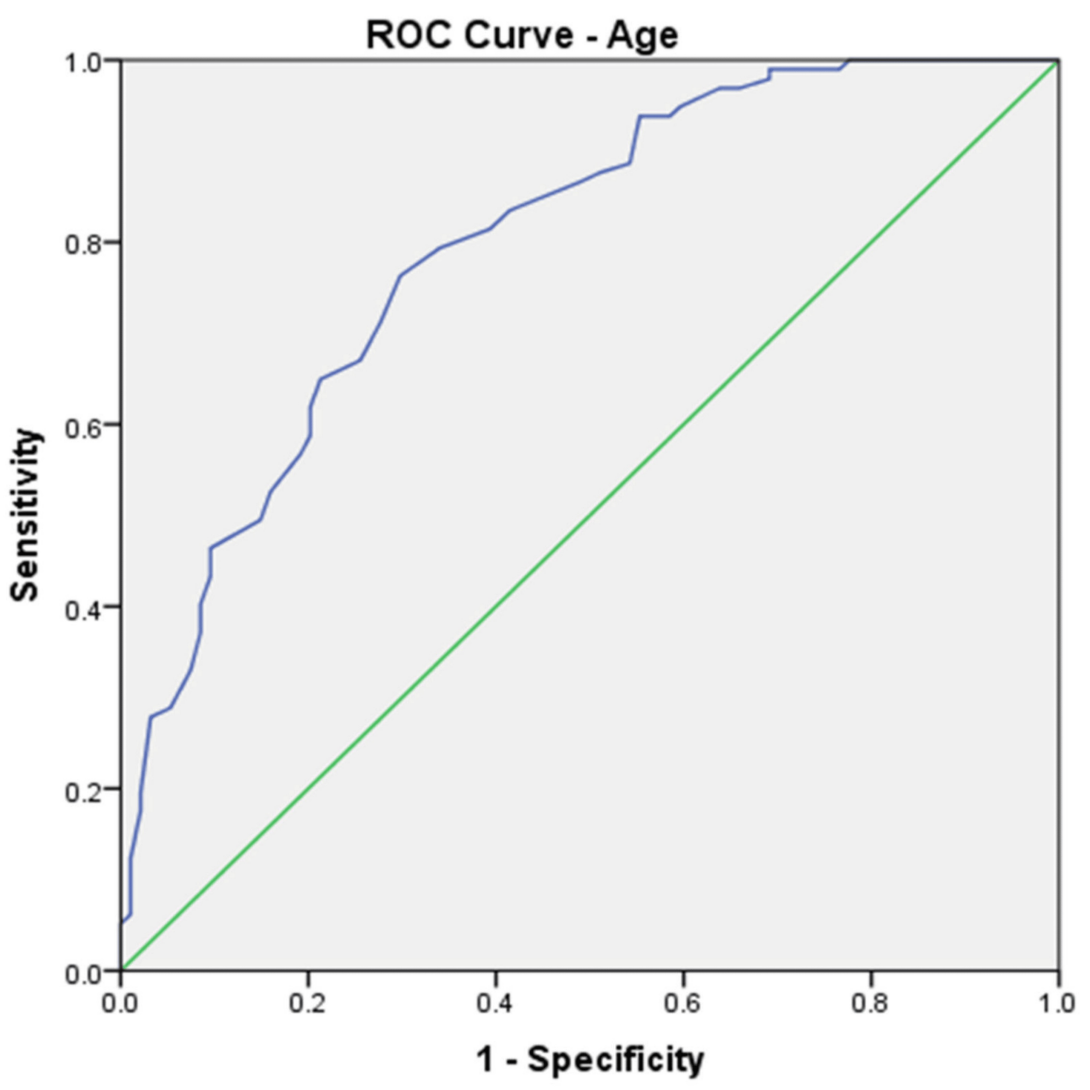
ROC curve for age predicting death in the analysed group (AUROC 0.799). ROC: receiver operating characteristic; AUROC: area under the receiver operating characteristic

**Figure 2:**
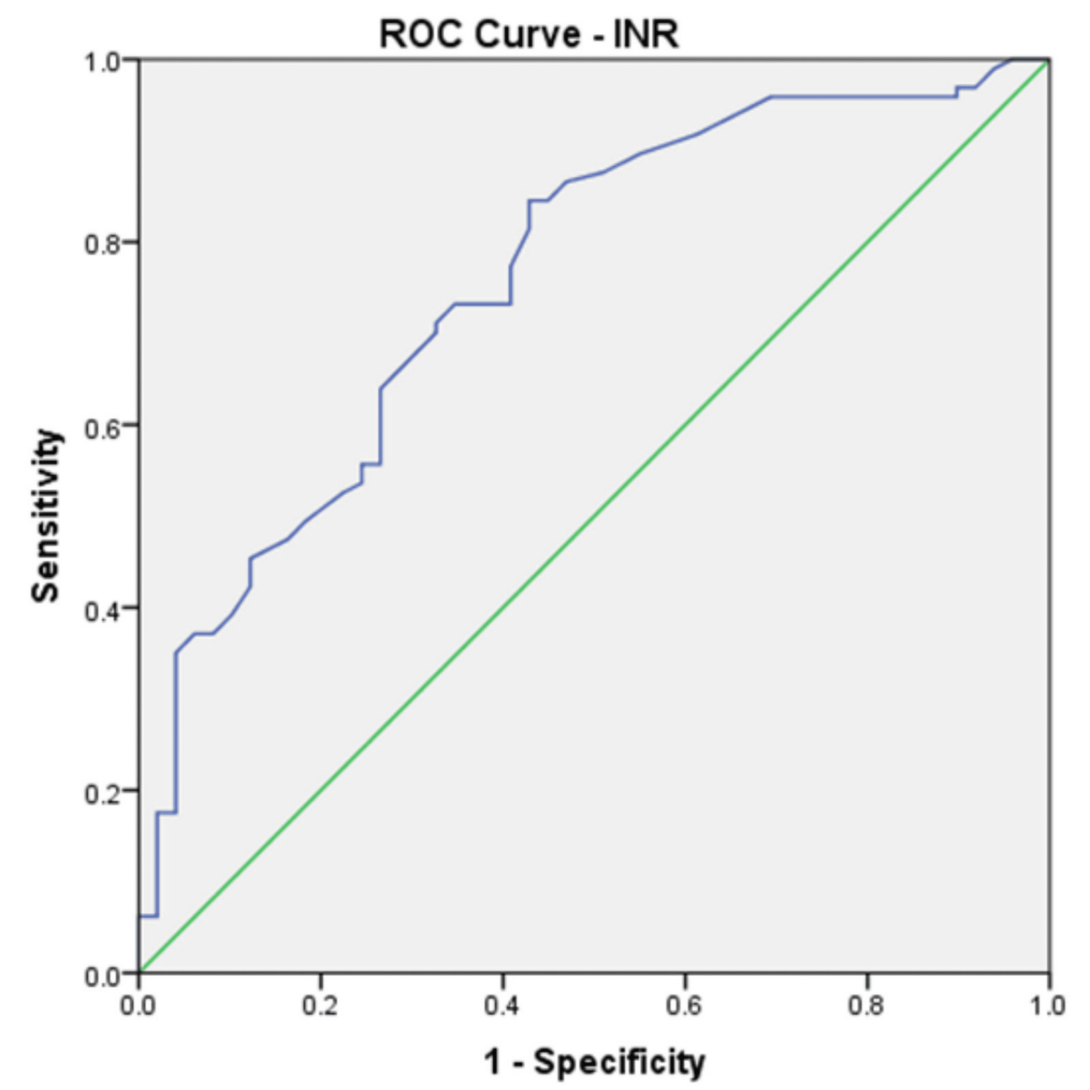
ROC curve for INR predicting death in the analysed group (AUROC 0.760). ROC: receiver operating characteristic; AUROC: area under the receiver operating characteristic; INR: international normalised ratio

We calculated ROC curves for all parameters that showed a significant difference at the means comparison test, and the results are shown in Table 6. ROC curves were calculated for each parameter using outcome death as the state variable. ROC curves confirmed that the four parameters identified by multivariate regression also have the highest AUROCs. Age (Figure 1) and INR (Figure 2) were the parameters with the highest AUROC.

**Table 6:** AUROC for tested variables as predictors for death in our group. Some parameters were not tested because the difference between groups was not significant. We considered a substantial AUROC of at least 0.600. The ROC curve shows the trade-off between sensitivity and specificity of a given model. AUROC is the measure of the classifier's ability to distinguish positive and negative classes. An AUROC of 0.5 indicates random results, while a value of 1 means that the model is perfect. We considered a substantial AUROC of at least 0.600. Systolic BP: systolic blood pressure; EGFR: estimated glomerular filtration rate; INR: international normalised ratio; CRP: C-reactive protein; CI: confidence interval

Variable	AUROC	Standard Error	Significance	95% CI
Age	0.799	0.031	0.000	0.737-0.860
Systolic BP	0.399	-	-	-
Leucocyte count	0.606	0.041	0.011	0.526-0.687
Haemoglobin	0.321	-	-	-
Creatinine	0.616	0.041	0.005	0.536-0.697
EGFR	0.343	-	-	-
Albumin	0.231	-	-	-
Potassium	0.355	-	-	-
INR	0.760	0.042	0.000	0.677-0.842
CRP	0.663	0.045	0.014	0.577-0.749
Fibrinogen	0.616	0.044	0.012	0.529-0.729

**Figure 3:**
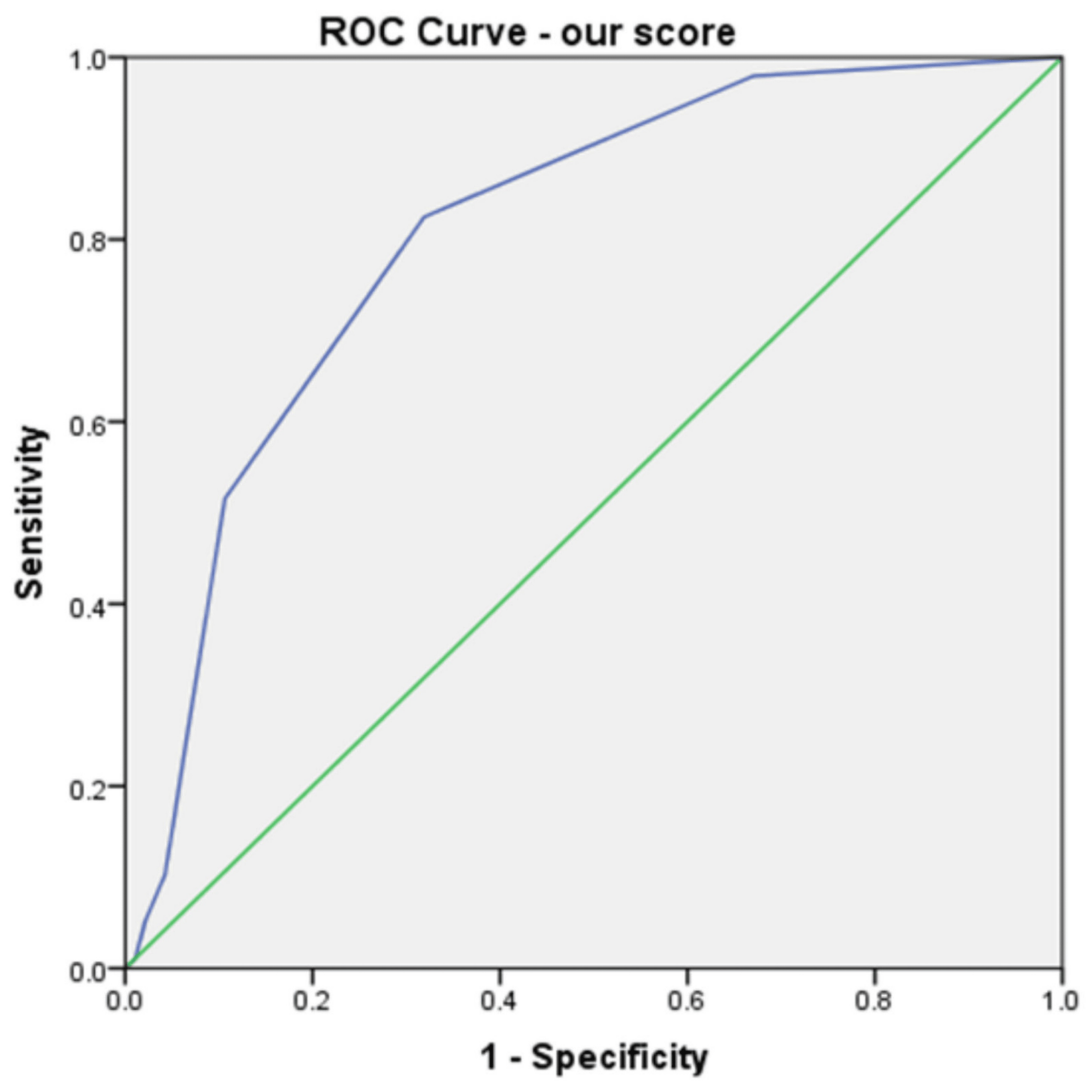
ROC curve for our new score predicting death in the analysed group (AUROC 0.807). ROC: receiver operating characteristic; AUROC: area under the receiver operating characteristic

Taking into account the independent-sample t-test, ROC curves, and multivariate analysis, only four factors can be considered independent predictors of death in our group: age, INR, CRP, and creatinine. Looking at the ROC curve coordinates, we selected the following cutoffs for the predictors of death, balancing the sensitivity and specificity of these: age higher than 70 years (80% sensitivity, 71% specificity), INR higher than 1.5 (77% sensitivity, 60% specificity), CRP higher than 5.7 mg/dl (80% sensitivity, 71% specificity), and creatinine higher than 2.1 mg/dl (50% sensitivity, 71% specificity). If we give each of them one point in a new score, we obtain a maximum of 4 points. This score has an excellent predictive value (Figure 3) in our group, with an AUROC of 0.828 (std. error 0.03, p<0.001, 95%CI 0.768-0.887), but needs validation in external studies. A scoring card for this score is shown in Table 7.

**Table 7:** Scoring table for a score that predicts death in CDI patients. INR: international normalised ratio; CRP: C-reactive protein; CDI: *Clostridioides difficile* infection

Result	0	1
Age (years)	<70	>70
INR	<1.5	>1.5
CRP (mg/dl)	<5.7	>5.7
Creatinine (mg/dl)	<2.1	>2.1

**Figure 4:**
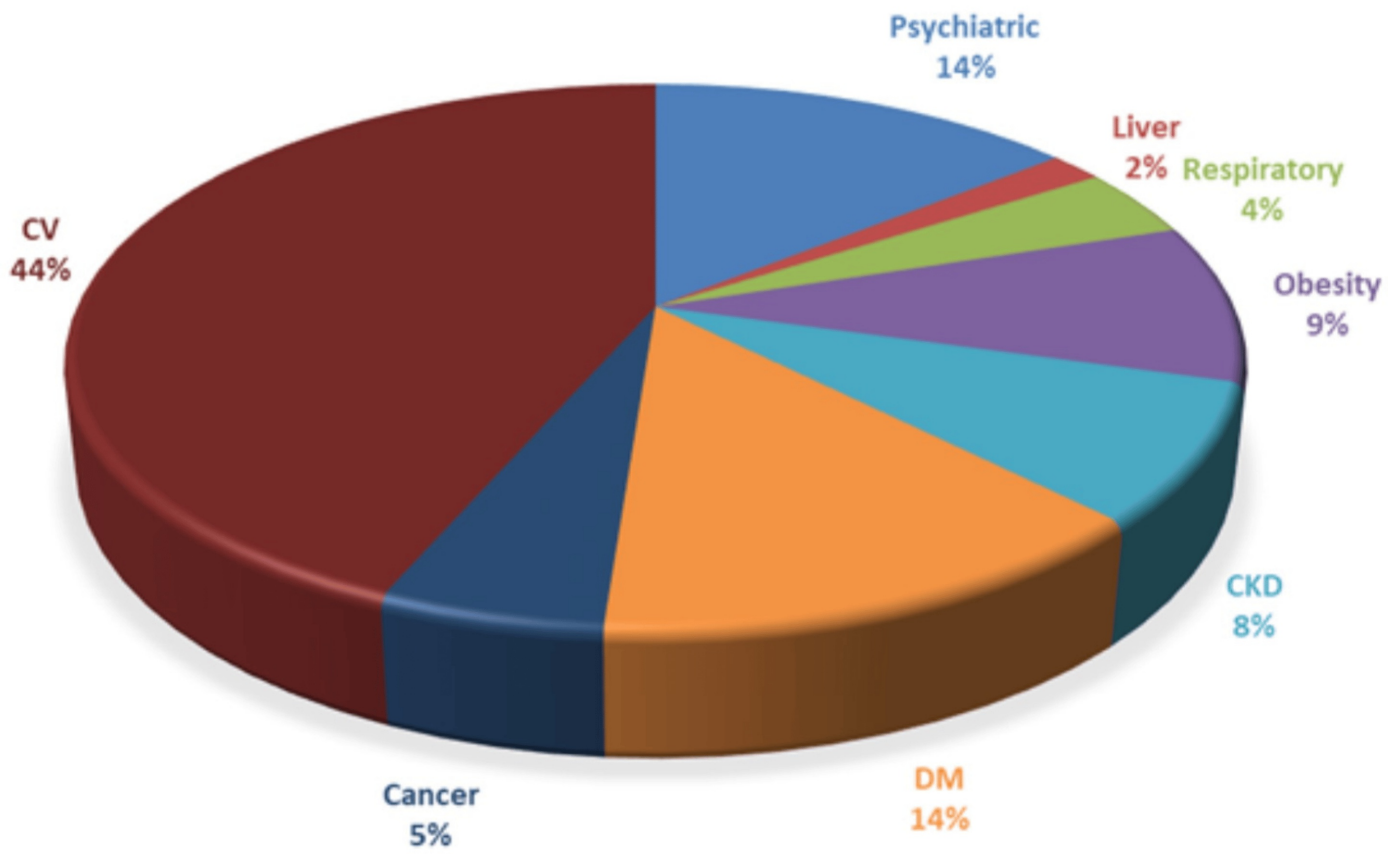
Comorbidities in the group of patients who died (N=97). CV: cardiovascular; DM: diabetes mellitus; CKD: chronic kidney disease

**Figure 5:**
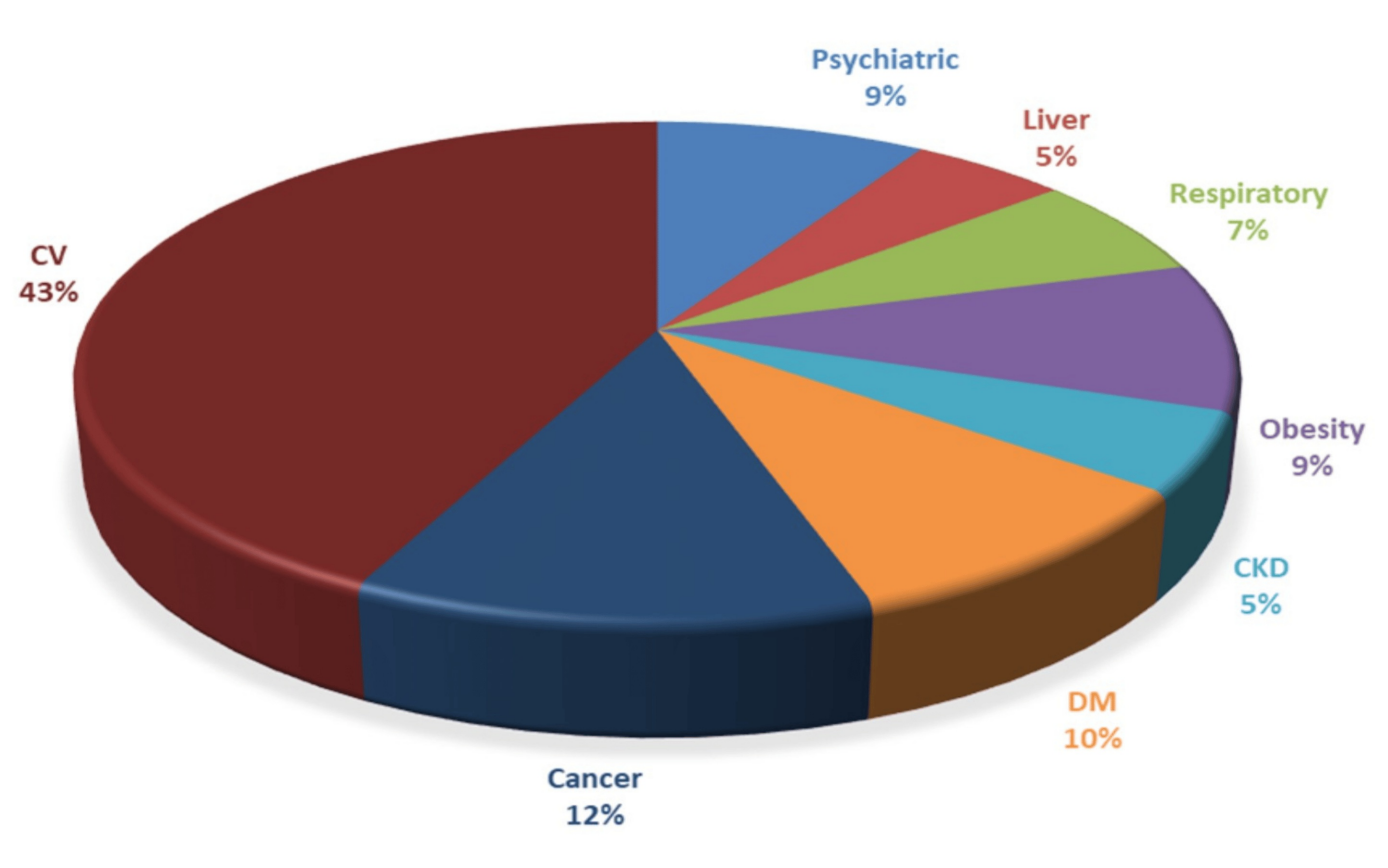
Comorbidities in the group of patients who survived (N=989). CV: cardiovascular; DM: diabetes mellitus; CKD: chronic kidney disease

Only two (2.1%) patients in the group of patients who died were free of any previous comorbidities. In contrast, 105 (10.6%) patients in the group of patients who did not die had no chronic comorbidities. The median number of comorbidities was 5.46 in the group of patients who died (0-15 concurrent diagnostics), and in the group of patients who survived, it was 5.47 (0-21 concurrent diagnostics). The types of comorbidities in each group are shown in Figures 4, 5.

The main differences between the two groups were that psychiatric diseases, diabetes mellitus, and chronic kidney diseases were more frequent in the group of patients who died. Liver diseases, respiratory tract diseases, and, surprisingly, cancer were more frequent in patients who survived. There are no differences between the frequencies of cardiovascular diseases and obesity between the two groups.

Not all patients used antibiotics in the previous 30 days before the current episode of CDI. Only 60 (61.9%) patients who died and 662 (66.9%) patients who survived used some antibiotics: 202 (31%) cefalosporins, 181 (27.4%) aminopenicillins, and 95 (14.3%) quinolones. We found no statistically significant differences between the types of antibiotics in the two groups. A total of 233 (21.5%) patients used PPI at the moment of hospitalisation and/or in the previous 30 days, with no statistical difference between the two groups.

Fifty-three (54.6%) patients who died were immobilised in bed, while in the group of patients who did not die, only 73 (7.4%) were in this situation (OR=14.97). The difference is statistically significant (p<0.001). Sixty-five (67%) patients who died and 616 (62.3%) of those who survived had a previous hospitalisation in the 30 days preceding hospitalisation (not a statistically significant difference).

## Discussion

In our group, the mean length of stay of CDI patients in the hospital is 15.4 (±13.09) days, while the mean length of stay of patients hospitalised with any morbidity in our infectious disease wards is 6.79 (±2.34). In the group of patients who died, the length of hospitalisation was 18.26 (±14.77) days, statistically significantly higher compared with 9.73 (± 6.77) in patients who did not die (p<0.001, 95% CI: 4.3-12.6). By comparison, other studies showed different durations of hospitalisation: a meta-analysis on almost one million patients in 10 years (2005-2015) in the United States showed an average length of stay of 11.1 days [30]; a similar length of stay was found in a Polish study covering two years (2011-2013), with a duration of 11 days [31]. Another 10-year study (2005-2015) in Australia found an average length of stay of 16.8-18.69 days [31]. Comparing the hospitalisation duration in CDI patients between countries and even hospitals is difficult because of more factors: different protocols, different characteristics of the patients, and different healthcare models. However, our study revealed that the length of stay in the case of the patients who died was longer than that of the patients who survived (which was expected because of the different severity of the diseases). Also, the average length of stay in CDI patients (in general and in both groups separately) is higher than that in the patients hospitalised for other morbidities.

Patients with CDI have a significantly higher risk of death than those hospitalised for other morbidities. As mentioned in the introduction, many studies already indicated this fact. In our group, the mortality was 8.39% for patients with CDI, in a period in which the mortality in our hospital for any other causes was 2.62%. Studies show mortality rates between 6% and 11% [18,19]. Mortality rates in ICU units are even higher, ranging from 20% to 37% [18]. Overall, there is a large variability between studies regarding the mortality rates, which is a multifactorial parameter, depending on how patients are selected, the study setting, comorbidities, and even case definition. A simple explanation would be that CDI is the appanage of already very sick patients (the median number of comorbidities in our group was 5.47, and the median Charlson score was 12.36). The most frequently encountered comorbidities in our study group were cardiovascular diseases (mainly stroke, ischaemic cardiac disease, and cardiac failure), psychiatric diseases (primarily dementia, schizophrenia, and different types of comportment disorders), malignancies (haematological or solid tumours), and diabetes mellitus. Psychiatric disorders, diabetes mellitus, and chronic kidney diseases were more frequent in the group of patients who died. In the group of patients who died, almost all but two (2.1%) patients had at least one comorbidity, while in the group of patients who survived, 105 (10.6%) of the patients were free of any comorbidity.

Not all patients who developed CDI used antibiotics before the current episode of CDI; specifically, only 60 (61.9%) of the patients who died and 662 (67%) of the patients who survived used antibiotics.

Significant differences exist between some of the parameters we included in the analysis. Still, the main predictors of death in our group are older age, high INR, high CRP, and high creatinine, which showed AUROCs of 0.799, 0.760, 0.663, and 0.616, respectively. Using these parameters, we created a simple score ranging from 0 to 4, which has a better predictive value than each of the factors involved separately. The AUROC for predicting death in our group for this score was 0.828. This score needs validation in external studies. In other studies, the predictors for mortality were age (older age has a higher risk), comorbidities, the severity of CDI (leucocytes and creatinine according to the above description), previous antibiotic use, delay in treatment, and recurrent CDI [20]. Some of these factors were also identified as increasing the rate of death in our group.

When interpreting the results of this analysis, we must also take into account the fact that its retrospective nature represents a study limitation that could have a potential impact on confounding variables. Another limitation of the present analysis is the fact that we could not provide data from colonoscopy; therefore, the relationships between CDI and diseases such as pseudomembranous colitis and inflammatory bowel disease (IBD) could not be established. We consider of interest further studies that analyse the relationships between CDI and these disorders, especially as biologic therapy used for IBD appears to be safe, with no red flags for *C. difficile* [32]. Furthermore, the lack of colonoscopy may impact severity assessments, highlighting the necessity for external validation of the risk score to confirm its efficacy across various contexts.

## Conclusions

In our cohort of CDI patients, the mortality rate was significantly higher than the mortality in patients hospitalised during the same period for any other diagnosis. The duration of hospitalisation in patients with CDI was more prolonged than in patients hospitalised for different diseases. The patients who eventually died had a significantly more extended hospitalisation than those who had a favourable outcome. Cardiovascular diseases were the most frequent underlying disease for both groups of patients. In the group of patients who died, psychiatric disorders were more frequently encountered, and a significantly higher percentage of patients who died were immobilised in bed compared with those with a favourable outcome.

Age, the previous number of CDI episodes, systolic BP, qSOFA, leucocyte count, haemoglobin, creatinine, albumin, potassium, INR, CRP, fibrinogen, procalcitonin, and ATLAS scores differed significantly between the patients who died and the patients with a favourable outcome. Multivariate regression identified four independent predictors of death in our cohort: older age, high INR, high CRP, and high creatinine. These can be used to calculate a risk score for an unfavourable outcome.
